# Battery Longevity in Modern Implantable Cardioverter‐Defibrillators and Cardiac Resynchronization Therapy‐Defibrillators

**DOI:** 10.1002/joa3.70175

**Published:** 2025-08-15

**Authors:** Kotaro Nishino, Taro Temma, Masaya Watanabe, Motoki Nakao, Masahiro Kawasaki, Kintaro Shimano, Kei Kawakami, Shota Saito, Jiro Koya, Daishiro Tatsuta, Hiroyuki Natsui, Takuya Koizumi, Takahide Kadosaka, Taro Koya, Kiwamu Kamiya, Toshiyuki Nagai, Toshihisa Anzai

**Affiliations:** ^1^ Department of Cardiovascular Medicine, Faculty of Medicine and Graduate School of Medicine Hokkaido University Sapporo Japan; ^2^ Department of Cardiovascular Medicine Hokko Memorial Hospital Sapporo Japan; ^3^ Department of Cardiovascular Medicine Otaru Kyokai Hospital Otaru Japan; ^4^ Department of Cardiovascular Medicine Hanaoka Seishu Memorial Hospital Sapporo Japan

**Keywords:** battery longevity, cardiac resynchronization therapy‐defibrillator, implantable cardioverter‐defibrillator, manufacturer

## Abstract

**Background:**

Battery longevity in high‐voltage devices (HVDs), specifically implantable cardioverter‐defibrillators (ICDs) and cardiac resynchronization therapy‐defibrillators (CRT‐Ds), is critical for reducing the frequency of generator replacements, minimizing procedural risks, and enhancing patient outcomes. Despite technological advancements, significant variability in battery performance remains among the major manufacturers. This study aimed to evaluate the battery longevity among ICDs and CRT‐Ds from the major manufacturers implanted at a single institution and identify the factors influencing battery depletion.

**Methods:**

We conducted a retrospective analysis of 353 patients implanted with HVDs (63 Abbott, 150 Boston Scientific, 140 Medtronic) at Hokkaido University Hospital between 2012 and 2021. Kaplan–Meier curves and Cox proportional hazards models were used to analyze the device longevity, with a primary endpoint of the time to battery depletion, defined by the elective replacement indicator. A multivariate analysis adjusted for the potential confounders.

**Results:**

Boston Scientific devices exhibited a significantly longer battery life than Abbott and Medtronic devices (*p* < 0.001), with a 6‐year replacement‐free survival of 99% for ICDs and 93% for CRT‐Ds. A multivariate analysis identified the device manufacturer, device type (ICD vs. CRT‐D), and ventricular pacing rate as independent predictors of battery depletion (*p* < 0.001).

**Conclusion:**

Battery longevity differed significantly by the manufacturer, which may influence device selection. Devices with a longer battery life may help reduce the replacement frequency and could potentially contribute to improved patient outcomes and cost‐effectiveness.

## Graphical abstract

A retrospective analysis of 353 patients implanted with HVDs between 2012 and 2021 revealed that 6‐year replacement‐free survival ranged from 99% to 100% for ICDs and 21%–93% for CRT‐Ds. Device manufacturer and ventricular pacing rate were identified as independent predictors of battery depletion. CRT‐D, cardiac resynchronization therapy‐defibrillator; HVD, high‐voltage device; ICD, implantable cardioverter‐defibrillator.

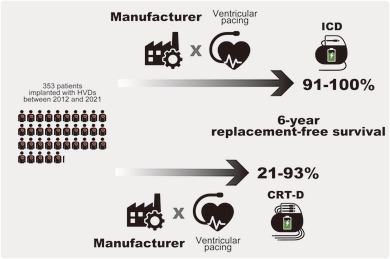

**Abbreviations**
ATP: anti‐tachycardia pacing
CIs: confidence intervals
CRT‐D: cardiac resynchronization therapy‐defibrillator
EMR: electronic medical record
HRs: hazard ratios
HVD: high‐voltage device
ICD: implantable cardioverter‐defibrillator

## 1 Introduction

High‐voltage devices (HVDs), including implantable cardioverter‐defibrillators (ICDs) and cardiac resynchronization therapy‐defibrillators (CRT‐Ds), are crucial for patients who have survived or are at high risk of sudden cardiac death due to ventricular arrhythmias, as demonstrated in numerous large‐scale studies [1, 2, 3, 4]. Although these HVDs improve the prognosis, they also present challenges, particularly the need for a generator replacement. This procedure is invasive and carries risks such as infection, bleeding, and lead fractures, whereas also imposing a financial burden on healthcare systems [5, 6, 7]. Although issues like recalls, corrosion, infections, and the need for device upgrades have become less common as reasons for generator replacements, battery depletion has emerged as the more prevalent reason for generator replacements [8].

In recent years, the battery longevity of HVDs has significantly improved across various types and clinical settings. However, no device is immune to battery depletion, making the extension of the battery life highly desirable. Prolonging the battery life could reduce the frequency of generator replacements, thereby minimizing the risk of complications and enhancing cost‐effectiveness and patient satisfaction. Several studies conducted in single‐center and multi‐center settings have indicated significant variability in the battery longevity of HVDs among the different device manufacturers [9, 10, 11]. However, the rapid pace of technological advancements in these devices suggests that previous findings may not fully apply to the current patient population.

This study aimed to evaluate the battery performance of HVDs from the major device manufacturers implanted in a contemporary cohort of patients at our institution.

## 2 Methods

### 2.1 Study Design

This retrospective observational study of HVD battery life was approved by the Ethics Committee of Hokkaido University Hospital (approval number: 023‐0198). As anonymized clinical data collected retrospectively were used, the requirement for informed consent for study participation was waived by the Ethics Committee in accordance with national ethical guidelines. Informed consent for the clinical implantation of HVDs was obtained from all patients as part of standard clinical practice. The primary endpoint of this analysis was the time from the device implantation to the device battery depletion, marked by the elective replacement indicator. In a sub‐analysis, patients were censored if the device was removed for reasons other than death or battery depletion such as infections, device and lead failures, heart transplants, and device upgrades. Patients were followed until the occurrence of an event or their last outpatient visit.

### 2.2 Patient Population

We enrolled 525 consecutive patients who underwent HVD implantations or replacements at Hokkaido University Hospital between January 1, 2012 and December 31, 2021. Demographic and clinical information was extracted from the electronic medical records (EMRs), and data on the date of the implantation, make and model, and all implanted leads were collected as the device baseline data. Detailed device information was obtained from the EMRs at the time of the implantation and during follow‐up visits. Device data included the pacing load, sensitivity, pacing threshold, pacing lead impedance for each lead, and history of defibrillation therapies (shocks or anti‐tachycardia pacing [ATP]) were also recorded. Although output voltage settings could be adjusted at the discretion of the attending physician based on individual clinical circumstances, they were generally programmed to twice the measured threshold voltage, consistent with our institutional protocol. Routine device interrogations were performed every 6 months to assess and, if necessary, adjust these settings. Furthermore, during the follow‐up period, remote monitoring was uniformly implemented across all manufacturers for patients who opted to consent to their use.

### 2.3 Statistical Analyses

Descriptive statistics for continuous variables are presented as means ± standard deviations or medians with interquartile ranges, depending on the distribution of the data. Categorical variables are summarized as frequencies and percentages. The Shapiro–Wilk test was used to assess the normality of the continuous variables. For normally distributed continuous variables, we used an analysis of variance or Student's *t*‐test to evaluate the differences between the groups. For non‐normally distributed continuous variables, the Wilcoxon rank‐sum test or Kruskal–Wallis test was employed. Categorical variables were compared using the Pearson chi‐square test or Fisher's exact test. Kaplan–Meier curves were constructed for the time from the device implantation to the device replacement due to battery depletion for all device manufacturers and were compared using the log‐rank test. To assess the differences in the time to battery depletion according to the device manufacturer, a Cox proportional hazards model was conducted, and hazard ratios (HRs) with 95% confidence intervals (CIs) were calculated. Covariates that could affect the time to battery depletion were included in a multivariate Cox proportional hazards model. For the comparisons between device manufacturers, we conducted pairwise post hoc comparisons using Cox proportional hazards models, and adjusted the significance level using the Bonferroni correction to account for multiple comparisons. Variables with a *p* value < 0.05 in the univariable analysis or with clinical relevance were included in the multivariable Cox proportional hazards model using a stepwise selection procedure. All statistical analyses were performed using JMP Pro 17.0.0 for Windows software (SAS Institute Inc., Cary, NC, USA). A two‐tailed *p* value of < 0.05 was considered statistically significant for all analyses. Where necessary, the significance level was adjusted for multiple comparisons using the Bonferroni correction.

## 3 Results

### 3.1 Patient Characteristics

**FIGURE 1:**
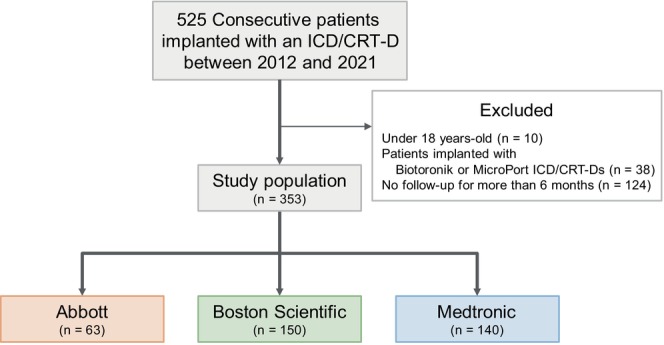
Study flow diagram. A total of 172 patients were excluded due to being under 18 years old at the time of the device implantation (*n* = 10), loss to follow‐up within 6 months (*n* = 124), or an implantation of devices from Biotronik or MicroPort (*n* = 38). The final study cohort included 353 patients: 63 with Abbott devices, 150 with Boston Scientific devices, and 140 with Medtronic devices. CRT‐D, cardiac resynchronization therapy‐defibrillator; ICD, implantable cardioverter‐defibrillator.

The study cohort consisted of 525 patients. Ten patients who were younger than 18 years at the time of the device implantation and 124 who were lost to follow‐up within 6 months of the device implantation were excluded. Additionally, 38 patients who underwent Biotronik or MicroPort device implantations were excluded due to the small number of those devices, which did not allow for an adequate analysis. However, the excluded patients are generally comparable to the included study population (Table S1). That left a final study cohort of 353 patients: 63 with devices from Abbott (formerly St. Jude Medical), 150 from Boston Scientific, and 140 from Medtronic (Figure 1). The specific models included from each manufacturer are summarized in Table S2. The baseline characteristics and device information are shown in Table 1. The cohort included 244 ICD recipients (69%) and 109 CRT‐D recipients (31%), with a median age of 62 (48–70) years, and 74% were male. There were significant differences in the right atrial threshold (Abbott: 0.75 [0.55–0.78] V, Boston Scientific: 0.60 [0.40–0.80] V, Medtronic: 0.60 [0.50–0.75] V, *p* = 0.017) and right atrial lead impedance (Abbott: 410 [369–475] ohms, Boston Scientific: 522 [444–580] ohms, Medtronic: 487 [418–566] ohms, *p* < 0.001) at the time of the implantation among the manufacturers. However, the other characteristics were similar across the groups.

**TABLE 1:** Baseline characteristics of the patients and device‐related information included in this analysis.

Variables	Overall (*n* = 353)	Abbott (*n* = 63)	Boston Scientific (*n* = 150)	Medtronic (*n* = 140)	*p*
Follow‐up, months	59 (34–83)	61 (43–102)	64 (35–89)	55 (32–75)	0.044
Age, years	62 (48–70)	56 (42–68)	61 (47–71)	64 (53–71)	0.016
Male	261 (74)	54 (86)	106 (71)	101 (72)	0.061
LVEF, %	39 (26–59)	44 (29–62)	40 (28–59)	36 (25–50)	0.212
NYHA class ≥III	77 (24)	13 (24)	31 (22)	33 (26)	0.820
*Comorbidities*					
Coronary artery disease	96 (28)	20 (33)	36 (24)	40 (29)	0.386
Diabetes mellitus	79 (23)	13 (22)	32 (22)	34 (25)	0.789
Hypertention	114 (33)	23 (38)	45 (30)	46 (34)	0.537
Atrial fibrillation	83 (25)	13 (22)	39 (27)	31 (24)	0.754
*Medications*					
Na channel blockers	14 (4)	5 (8)	5 (3)	4 (3)	0.224
Beta‐blockers	258 (74)	38 (64)	105 (71)	115 (82)	0.015
Amiodarone	132 (38)	24 (40)	52 (36)	56 (40)	0.744
Ca channel blockers	43 (13)	7 (12)	16 (11)	20 (14)	0.700
Spironolactone	147 (42)	26 (43)	53 (36)	68 (49)	0.088
Digitalis	10 (3)	2 (3)	5 (3)	3 (2)	0.761
*Device type*					
ICD	244 (69)	47 (75)	106 (71)	91 (65)	0.338
CRT‐D	109 (31)	16 (25)	44 (29)	49 (35)	0.338
*Indication*					
Primary prevention	153 (45)	22 (37)	66 (45)	65 (48)	0.375
Secondary prevention	189 (55)	37 (63)	82 (55)	70 (52)	0.375
*Parameters*					
RA threshold, V	0.60 (0.50–0.76)	0.75 (0.55–0.78)	0.60 (0.40–0.80)	0.60 (0.50–0.75)	0.017
RA pulse width, ms	0.40 (0.40–0.50)	0.40 (0.40–0.50)	0.40 (0.40–0.50)	0.40 (0.40–0.50)	0.566
RA impedance, ohms	494 (413–561)	410 (369–475)	522 (444–580)	487 (418–566)	< 0.001
RV threshold, V	0.70 (0.50–1.00)	0.70 (0.50–1.00)	0.60 (0.40–0.98)	0.75 (0.50–1.00)	0.111
RV pulse width, ms	0.40 (0.40–0.50)	0.40 (0.40–0.50)	0.40 (0.40–0.50)	0.40 (0.40–0.50)	0.149
RV impedance, ohms	494 (420–594)	510 (430–620)	478 (420–582)	498 (415–588)	0.466
LV threshold, V	1.00 (0.70–1.29)	0.84 (0.75–1.00)	1.00 (0.80–1.30)	0.89 (0.50–1.25)	0.171
LV pulse width, ms	0.40 (0.40–0.50)	0.45 (0.40–0.50)	0.40 (0.40–0.40)	0.40 (0.40–0.50)	0.064
LV impedance, ohms	532 (420–680)	625 (490–783)	548 (404–689)	512 (417–660)	0.371

*Note:* Continuous variables are presented as the mean ± standard deviation if normally distributed and median (inter‐quartile range) if not normally distributed. Categorical variables are presented as the number of patients (%).

Abbreviations: CRT‐D, cardiac resynchronization therapy‐defibrillator; ICD, implantable cardioverter‐defibrillator; LV, left ventricle; LVEF, left ventricular ejection fraction; NYHA, New York Heart Association; RA, right atrium; RV, right ventricle.

### 3.2 Battery Depletion During the Follow‐Up

**FIGURE 2:**
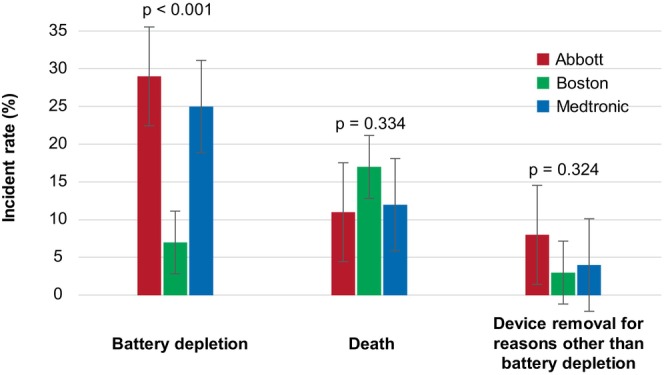
Incident rates occurring during the follow‐up period. During the follow‐up, 14% of the patients died before the device replacement, with no significant differences between the manufacturers. Overall, 22% of the patients had their device replaced or removed for any reason, with 18% due to battery depletion. Boston Scientific showed a significantly lower replacement rate due to battery depletion than Abbott or Medtronic (Abbott: 29%, Boston Scientific: 7%, Medtronic: 25%, *p* < 0.001). There were no significant differences between the manufacturers in the device replacements or removals for reasons other than death or battery depletion.

The median follow‐up period was 59 (34–83) months, with slight differences observed between the manufacturers (Abbott: 61 [43–102] months, Boston Scientific: 64 [35–89] months, Medtronic: 55 [32–75] months, *p* = 0.044, Table 1). During the follow‐up, 50 patients (14%) died before the device replacement, and 79 (22%) had their device replaced or removed for various reasons. Of those, 63 patients (18%) underwent device replacements due to battery depletion, with a significantly lower replacement rate for Boston Scientific devices (Abbott: 18 patients [29%], Boston Scientific: 10 patients [7%], Medtronic: 35 patients [25%], *p* < 0.001). Other reasons for the replacement or device removal included heart transplantations (8 cases), lead failures (5 cases), device upgrades (2 cases), and an infection (1 case). There were no significant differences between the manufacturers in the rates of device replacements or removals for reasons other than death or battery depletion prior to the device replacement (Figure 2).

### 3.3 Evaluation of the Device Longevity Among the Manufacturers

**FIGURE 3:**
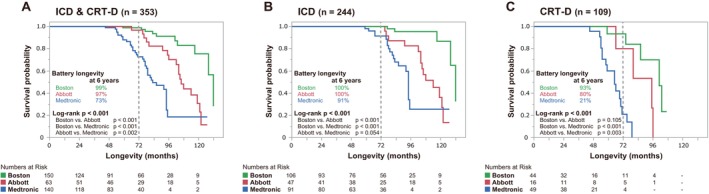
Kaplan–Meier curves showing the battery longevity by manufacturer. (A) The estimates for all devices (ICDs and CRT‐Ds combined); (B) the estimates for ICDs only; and (C) the estimates for CRT‐Ds only. Significant differences in the device longevity were observed across the manufacturers for both ICDs and CRT‐Ds (overall log‐rank test, all *p* < 0.001). The 6‐year survival rates for ICDs were 100% for Abbott, 100% for Boston Scientific, and 91% for Medtronic, with Boston Scientific having a significantly longer survival than the others. For CRT‐Ds, the 6‐year survival rates were 80% for Abbott, 93% for Boston Scientific, and 21% for Medtronic, with both Abbott and Boston Scientific demonstrating a significantly longer survival than Medtronic. CRT‐D, cardiac resynchronization therapy‐defibrillator; ICD, implantable cardioverter‐defibrillator.

**FIGURE 4:**
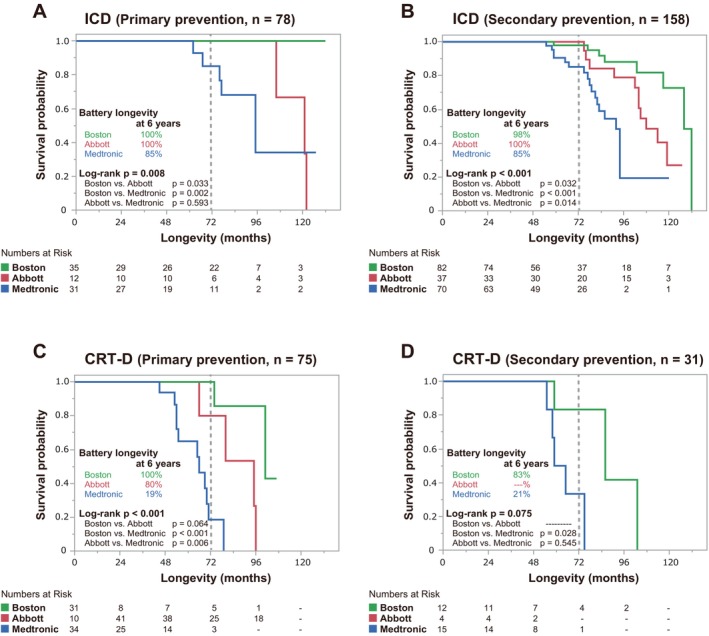
Kaplan–Meier curves showing the battery longevity by manufacturer categorized by primary and secondary prevention strategies. (A) ICDs in the primary prevention group exhibited significant differences in the device longevity between the manufacturers (overall log‐rank test, *p* = 0.008). (B) ICDs in the secondary prevention group also exhibited significant differences between the manufacturers (overall log‐rank test, *p* < 0.001). (C) CRT‐Ds in the primary prevention group exhibited significant differences between the manufacturers (overall log‐rank test, *p* < 0.001). (D) CRT‐Ds in the secondary prevention group did not exhibit statistically significant differences in the device longevity between the manufacturers. CRT‐D, cardiac resynchronization therapy‐defibrillator; ICD, implantable cardioverter‐defibrillator.

Figure 3 presents Kaplan–Meier estimates of the time to device replacement due to battery depletion, revealing significant differences in the device longevity across the manufacturers in the overall (Figure 3A), ICD‐only (Figure 3B), and CRT‐D‐only analysis (Figure 3C) groups (overall log‐rank test, all *p* < 0.001). The 6‐year survival rates for ICDs were 100% for Abbott, 100% for Boston Scientific, and 91% for Medtronic, with Boston Scientific showing a significantly longer survival than the other two (Boston Scientific vs. Abbott log‐rank, *p* < 0.001; Boston Scientific vs. Medtronic, log‐rank *p* < 0.001). For CRT‐Ds, the 6‐year survival rates were 80% for Abbott, 93% for Boston Scientific, and 21% for Medtronic, with Abbott and Boston Scientific demonstrating a significantly longer survival than Medtronic (Boston Scientific vs. Medtronic log‐rank *p* < 0.001, Abbott vs. Medtronic log‐rank *p* = 0.003). Overall, the median device life was 121 (95–132) months for ICDs and 75 (65–96) months for CRT‐Ds. Figure 4 displays the Kaplan–Meier estimates of the battery longevity for ICDs and CRT‐Ds, categorized by primary and secondary prevention strategies. In the ICD group, significant differences in the device longevity were observed between manufacturers in both the primary prevention (overall log‐rank test, *p* = 0.008) (Figure 4A) and secondary prevention groups (overall log‐rank test, *p* < 0.001) (Figure 4B). Similarly, in the CRT‐D group, significant differences in the device longevity were noted between the manufacturers in the primary prevention group (overall log‐rank test, *p* < 0.001) (Figure 4C). However, in the secondary prevention group, whereas there was a tendency for differences in the device longevity between the manufacturers, those differences were not statistically significant (Figure 4D).

### 3.4 Comparison of the Device Parameters and Therapeutic Interventions at the Last Follow‐Up

Table 2 presents the pacing threshold, lead impedance, pacing rate, and history of therapeutic intervention with shocks and ATP at the last visit. Abbott devices tended to have a lower right atrial lead impedance at the last visit compared to the other manufacturers (Abbott: 320 [300–355] ohms, Boston Scientific: 473 [421–527] ohms, Medtronic: 448 [380–499] ohms, *p* < 0.001), and also tended to have a lower upper pacing rate (Abbott: 118 [103–120] b.p.m., Boston Scientific: 130 [120–130] b.p.m., Medtronic: 120 [120–130] b.p.m., *p* = 0.006). The lower pacing rate differed significantly among the three groups (Abbott: 40 [40–60] b.p.m., Boston Scientific: 50 [40–60] b.p.m., Medtronic: 50 [40–60] b.p.m., *p* = 0.026), although no significant pairwise differences were observed between the manufacturers. Other device parameters were comparable across the manufacturers. The percentage of patients with a history of shocks and ATP, the total number of shocks and ATP, and the total energy of the shocks did not differ significantly between the manufacturers.

**TABLE 2:** Device parameters and treatment intervention history immediately prior to the device replacement due to battery depletion.

Variables	Overall (*n* = 63)	Abbott (*n* = 18)	Boston Scientific (*n* = 10)	Medtronic (*n* = 35)	*p*
*Parameters*					
RA threshold, V	0.70 (0.50–1.00)	0.63 (0.50–0.94)	0.80 (0.70–0.90)	0.63 (0.50–1.00)	0.555
RA pulse width, ms	0.40 (0.40–0.40)	0.40 (0.40–0.40)	0.40 (0.40–0.40)	0.40 (0.40–0.40)	0.724
RA impedance, ohms	418 (342–494)	320 (300–355)	473 (421–527)	448 (380–499)	< 0.001
RA pacing burden, %	25 (1–88)	18 (1–61)	1 (0–100)	54 (1–87)	0.844
RV threshold, V	0.88 (0.75–1.18)	0.80 (0.75–1.00)	1.00 (0.88–1.03)	0.75 (0.75–1.63)	0.402
RV pulse width, ms	0.40 (0.40–0.40)	0.40 (0.40–0.40)	0.40 (0.40–0.40)	0.40 (0.40–0.40)	0.815
RV impedance, ohms	441 ± 131	407 ± 149	460 ± 120	453 ± 124	0.432
RV pacing burden, %	93 (1–99)	1 (1–96)	77 (1–100)	98 (0–100)	0.107
LV threshold, V	1.90 ± 1.16	0.44 ± 0.09	2.25 ± 0.87	1.99 ± 1.18	0.161
LV pulse width, ms	0.40 (0.40–0.40)	0.50 (0.40–0.60)	0.40 (0.33–1.23)	0.40 (0.40–0.40)	0.661
LV impedance, ohms	539 ± 160	570 ± 127	589 ± 124	524 ± 174	0.754
LV pacing burden, %	99 (93–100)	98 (97–99)	100 (89–100)	n.a.	0.767
Lower pacing rate, b.p.m.	50 (40–60)	40 (40–60)	50 (40–60)	50 (40–60)	0.026
Upper pacing rate, b.p.m.	120 (120–130)	118 (103–120)	130 (120–130)	120 (120–130)	0.006
*Therapies*					
Patients receiving any shocks	18 (29)	5 (28)	3 (30)	10 (29)	1.000
Number of shocks	0 (0–1)	0 (0–1)	0 (0–2)	0 (0–1)	0.967
Total energy of shock	0 (0–30)	0 (0–30)	0 (0–52)	0 (0–20)	0.950
Patients receiving any ATPs	17 (27)	6 (33)	2 (20)	9 (26)	0.792
Number of ATPs	0 (0–1)	0 (0–2)	0 (0–0)	0 (0–1)	0.694

*Note:* Continuous variables are presented as the mean ± standard deviation if normally distributed and median (inter‐quartile range) if not normally distributed. Categorical variables are presented as the number of patients (%).

Abbreviations: ATP, anti‐tachycardia pacing; LV, left ventricle; RA, right atrium; RV, right ventricle.

### 3.5 Predictors of Battery Depletion

A univariate analysis identified several predictors of battery depletion including the device manufacturer (Boston Scientific vs. Abbott: HR 0.24, 95% CI 0.10–0.55, *p* = 0.001, Boston Scientific vs. Medtronic: HR 0.09, 95% CI 0.04–0.20, *p* < 0.001, Abbott vs. Medtronic: HR 0.38, 95% CI 0.21–0.71, *p* = 0.002), type of device (ICD vs. CRT‐D: HR 0.15, 95% CI 0.09–0.26, *p* < 0.001), atrial pacing rate (per 10%, HR 1.09, 95% CI 1.01–1.16, *p* = 0.023), ventricular pacing rate (per 10%, HR 1.25, 95% CI 1.18–1.34, *p* < 0.001), and number of ATP activations (per 1 time, HR 1.01, 95% CI 1.00–1.02, *p* = 0.011). The number of shock activations and total shock energy, however, were not associated with battery depletion. A multivariate analysis confirmed that the device manufacturer (Boston Scientific vs. Abbott: HR 0.13, 95% CI 0.04–0.40, *p* < 0.001, Boston Scientific vs. Medtronic: HR 0.02, 95% CI 0.00–0.06, *p* < 0.001, Abbott vs. Medtronic: HR 0.12, 95% CI 0.04–0.34, *p* < 0.001), device type (ICD vs. CRT‐D: HR 0.23, 95% CI 0.09–0.60, *p* = 0.003), and ventricular pacing rate (per 10%, HR 1.16, 95% CI 1.04–1.28, *p* = 0.011) were independent predictors of battery depletion (Table 3).

**TABLE 3:** Univariate and multivariate analysis of the factors associated with battery depletion.

Variable	Univariable analysis			Multivariable analysis		
	HR	95% CI	*p*	HR	95% CI	*p*
Boston vs. Abbott	0.24	0.10–0.55	0.001	0.13	0.04–0.40	< 0.001
Boston vs. Medtronic	0.09	0.04–0.20	< 0.001	0.02	0.00–0.06	< 0.001
Abbott vs. Medtronic	0.38	0.21–0.71	0.002	0.12	0.04–0.34	< 0.001
ICD vs. CRT‐D	0.15	0.09–0.26	< 0.001	0.23	0.09–0.60	0.003
Percentage of atrial pacing, per 10%	1.09	1.01–1.16	0.023	1.07	0.99–1.16	0.078
Percentage of ventricular pacing, per 10%	1.25	1.18–1.34	< 0.001	1.16	1.04–1.28	0.011
Threshould of right ventricular pacing, per 1 V	1.00	0.62–1.44	0.983			
Number of shocks, per 1 time	1.00	0.91–1.06	0.935			
Total energy of shocks, per 10 joule	1.02	0.98–1.05	0.292			
Number of ATPs, per 1 time	1.01	1.00–1.02	0.011	1.00	0.99–1.01	0.334

Abbreviations: ATP, antitachycardia pacing; CI, confidence interval; CRT‐D, cardiac resynchronization‐therapy defibrillator; HR, hazard ratio; ICD, implantable cardioverter‐defibrillator.

## 4 Discussion

In this study, we conducted a comprehensive evaluation of the battery performance among ICDs and CRT‐Ds from the major manufacturers, focusing on a contemporary cohort of patients at our institution. Our findings revealed a significant variability in the battery longevity between the devices, with Boston Scientific demonstrating a superior performance compared to Abbott and Medtronic. Those differences persisted even after adjusting for the potential confounders, suggesting that manufacturer‐specific factors play a crucial role in battery longevity. The overall device life was approximately 10 years for ICDs and just over 6 years for CRT‐Ds, showing significant differences in device longevity among the three major manufacturers, regardless of the defibrillator therapy burden.

Poli et al. [8] collected records of 29 158 ICD/CRT‐D devices that underwent replacements during the 10‐year period from 2007 to 2016 and found that the median device life ranged from 55.0 to 87.6 months in the single‐chamber ICD group, 48.3–77.0 months in the dual‐chamber ICD group, and 36.4–61.5 months in the CRT‐D group. Although our study included records of ICD/CRT‐D devices implanted during the 10‐year period from 2012 to 2021, it suggests that the ICD/CRT‐D battery life has been further extended in more contemporary cohorts, likely reflecting the latest technological advancements in device design and battery management.

The disparity between the survival of patients with ICD/CRT‐D implants and the useful life of the devices, often shorter than the patient survival, remains a significant concern [7]. Notably, during our follow‐up period, only Boston Scientific devices had a replacement rate due to battery depletion lower than the mortality rate. Williams et al. [12] previously reported in 2020 that the CRT‐D battery life exceeded the patient survival in a heart failure cohort with a reduced left ventricular ejection fraction. That finding underscores the importance of selecting devices with a longer battery life, particularly for patients requiring long‐term device therapy. The superior performance of Boston Scientific devices may reduce the frequency of generator replacements, thereby minimizing risks such as infection and lead damage and reducing healthcare costs, especially in younger patients.

In our cohort, the primary cause of ICD/CRT‐D replacements was battery depletion. Advances in the surgical techniques and device technology, along with the widespread use of remote monitoring, may have contributed to reduced complication rates and fewer device replacements due to appropriate consideration of device indications and advances in medical therapy. Factors affecting the battery life can be broadly categorized into the energy consumed by the device and the energy available from the battery. Consistent with most previous studies, our analysis identified the device manufacturer, CRT‐Ds (versus ICDs), and the ventricular pacing rate as independent predictors of battery depletion, whereas tachycardia therapies such as shocks and ATP were not associated with battery depletion [7].

The energy available in a battery is generally determined by its overall capacity, chemistry, and internal design. Differences in the battery life between manufacturers can be attributed to variations in the construction and design. For example, the ENDURALIFE battery (Boston Scientific, St. Paul, MN, USA) uses Li/MnO_2_ chemistry and a stacked plate structure inside the battery to maximize the power capacity and energy density while maintaining device thinness and minimizing the internal resistance [13]. Additionally, the optimized output efficiency, which is not affected by a low battery voltage or pacing output settings, and a circuit design that reduces the power consumption are expected to extend the battery life. In contrast to Li/SVO and Li/SVO‐CFx hybrid batteries—which typically experience a drop in voltage to approximately 2.6 V and a sharp rise in internal resistance once around 70% of the battery has been depleted, prompting the setting of the elective replacement indicator at that point—Li/MnO_2_‐based ENDURALIFE batteries can sustain a voltage above 2.8 V and maintain relatively stable internal resistance throughout most of the discharge cycle. This superior discharge profile allows the ERI to be set at a higher depletion threshold, around 90%, whereas still ensuring an adequate safety margin [13].

Our study highlights the need for further research to explore the underlying mechanisms that contribute to the observed differences in battery performance. Although our multivariate analysis identified the manufacturer, device type, and ventricular pacing rate as independent predictors of battery depletion, the specific technological innovations or design choices that drive those differences remain unclear. Future studies should aim to dissect those factors to improve device development and optimize patient outcomes.

## 5 Limitations

This study had several limitations that should be considered when interpreting the results. First, as a retrospective study conducted at a single institution, the findings may not be generalizable to other institutions. In particular, differences in institutional practice patterns, device programming strategies, and physician preferences across institutions and regions may limit the external applicability of our results. However, by including all consecutive patients who underwent ICD or CRT‐D implantations or replacements at our institution, we minimized the selection bias and provided a reflection of the real‐world clinical data. Second, device measurements were not collected throughout the entire period of the device function, and not all measurements were included in the analysis. Despite this, we believe that the measurements taken at the last visit were a reliable surrogate for the device's overall performance. Additionally, while the pacing output was not directly recorded, it was set to twice the measured pacing threshold, which we consider a reliable proxy for the pacing output. Third, although we collected detailed data on both the devices and patients, there may have been unmeasured confounders that were not accounted for in the analysis. Factors such as patient adherence to follow‐up visits, lifestyle changes, and comorbidities could impact the device performance and longevity but were not fully captured in this study. In addition, specific data on atrial ATP therapy, which may influence battery consumption particularly in Medtronic devices, were not systematically available and could not be analyzed in this study. Fourth, devices from Biotronik or MicroPort were excluded due to an insufficient number of cases, limiting our ability to analyze and discuss the characteristics of those manufacturers' devices. Future studies should aim to address these limitations by including a more diverse patient population, considering additional confounding factors, and continuously updating the findings to reflect the latest advancements in device technology.

## 6 Conclusion

In a contemporary cohort, we found that the median battery life of ICDs and CRT‐Ds was approximately 10 and 6 years, respectively, with significant differences between the manufacturers. Those differences may suggest the importance of device selection, as frequent replacements can lead to the risk of complications and an increased healthcare cost. By prioritizing devices with longer battery lives and understanding the factors influencing battery depletion, healthcare providers may be able to improve patient outcomes and promote cost‐effectiveness. These findings may also inform health policy decisions and resource allocation strategies by highlighting the long‐term economic impact of device longevity.

## Ethics Statement

This study was approved by the Ethics Committee of Hokkaido University Hospital (Local approval number: 023‐0198) and was conducted according to the principles outlined in the Declaration of Helsinki.

## Consent

Informed consent was obtained from all individual participants included in the study.

## Conflicts of Interest

Dr. Anzai received clinical research grants from Abbott Medical Japan LLC., Japan Lifeline Co. Ltd., and Boston Scientific Co. Ltd., and scholarship funds from Medtronic Japan Co. Ltd.

## Supporting information


**Data S1:** joa370175‐sup‐0001‐supinfo.docx.

## Data Availability

The data underlying this article will be shared upon reasonable request from the corresponding authors.
