# Supplementary material for: Battery Longevity in Modern Implantable Cardioverter‐Defibrillators and Cardiac Resynchronization Therapy‐Defibrillators
Source: J Arrhythm. 2025 Aug 15;41(4):e70175. doi: 10.1002/joa3.70175 (PMC12355725; doi:10.1002/joa3.70175)
Supplement: Supplementary file 1 — Data S1: joa370175‐sup‐0001‐supinfo.docx. [file JOA3-41-e70175-s001.docx]

**Supplemental Material**

This supplemental file is intended for publication as an online data supplement.

**Table S1: Patient characteristics classified by inclusion and exclusion status.**

Continuous variables are presented as the mean ± standard deviation if normally distributed and median (inter-quartile range) if not normally distributed. Categorical variables are presented as the number of patients (%).

CRT-D, cardiac resynchronization therapy-defibrillator; ICD, implantable cardioverter-defibrillator; LV, left ventricle; LVEF, left ventricular ejection fraction; NYHA, New York Heart Association; RA, right atrium; RV, right ventricle.

**Table S2: List of ICD and CRT-D models by manufacturer, release year, and frequency of use in the study cohort.**

CRT-D, cardiac resynchronization therapy-defibrillator; ICD, implantable cardioverter-defibrillator.
